# Homometallic Intervalence Charge Transfer Band of Co(II/III) Induced by Phase Transitions in a Heterometallic Co–W Charge Transfer Photomagnet

**DOI:** 10.1002/smsc.202500469

**Published:** 2025-11-11

**Authors:** Kazuki Nakamura, Koji Nakabayashi, Yuito Nosaka, Wakano Ota, Takashi Kikuchi, Shin‐ichi Ohkoshi

**Affiliations:** ^1^ Department of Chemistry School of Science The University of Tokyo 7‐3‐1 Hongo, Bunkyo‐ku Tokyo 113‐0033 Japan; ^2^ DYNACOM IRL2015 University of Tokyo CNRS Universite de Rennes 7‐3‐1 Hongo, Bunkyo‐ku Tokyo 113‐0033 Japan; ^3^ Rigaku Corporation 3‐9‐12, Matsubara‐cho Akishima‐shi Tokyo 196‐8666 Japan

**Keywords:** charge‐transfer phase transition, cyanido‐bridged assemblies, intervalence charge transfer, mixed‐valence chemistry, photomagnetism

## Abstract

A photomagnet, Co_8_[W(CN)_8_]_5_Cl·(pyrazine)_11_·21H_2_O, exhibiting Class II mixed valency due to homometallic intervalence charge transfer (IVCT) between Co^II^ and Co^III^ centers, is synthesized. The compound features a 3D cyanido‐bridged CoW coordination network composed of two crystallographically independent Co sites (Co1 and Co2) and one W site. Rectangular tubular structures formed by Co1‐W bridges are further crosslinked by the Co2 sites. Defects in the [W(CN)_8_] sites enable the formation of pyrazine bridges between the Co1 sites. Upon cooling, the compound undergoes a two‐step thermal phase transition, attributed to heterometallic charge‐transfer‐induced spin transitions between high‐ and low‐spin electronic states. The first‐step charge transfer (CT) phase transition leads to the formation of homometallic [Co^II^–pyrazine–Co^III^] bridges, producing a near‐infrared IVCT band at 2300 nm. Photoirradiation at 785 nm at 3 K induces a transition to a photoinduced (PI1) phase. The PI1 phase also shows a homometallic IVCT band due to the emergence of the [Co^II^–pyrazine–Co^III^] state. Subsequent photoirradiation at 532 nm to the PI1 phase induces a transition to the paramagnetic photoinduced (PI2) phase. This study demonstrates the modulation of electronic states in a phase transition material and a photomagnet, enabled by homo‐ and heterometallic CT processes.

## Graphical abstract

A photoinduced phase transition via heterometallic intervalence charge transfer (IVCT) between Co and W induces homometallic IVCT between Co^II^ and Co^III^ in a cyanido‐bridged Co–W photomagnet.

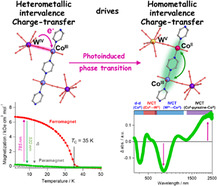

## 1 Introduction

Molecule‐based materials that undergo phase transitions and structural changes in response to external stimuli and environmental conditions have been extensively studied across various research fields due to their promising functionalities in medical, biochemical, and materials science.^[^ ^1^, ^2^, ^3^, ^4^, ^5^, ^6^, ^7^, ^8^ ^]^ These materials offer advantages in design flexibility, which enable optimization and the realization of multiple functionalities.^[^ ^9^, ^10^, ^11^, ^12^, ^13^ ^]^ In functional molecule‐based materials, mixed valency arising from charge transfer (CT) between electron donors and acceptors is a classic and crucial factor underlying functionalities such as conductivity, catalysis, and magnetism.^[^ ^14^, ^15^, ^16^, ^17^, ^18^, ^19^, ^20^, ^21^ ^]^ Mixed‐valence systems are grouped into three classes based on the degree of electronic interactions according to the Robin‐Day classification: essentially no interaction (Class I), weak to moderate interaction (Class II), and strong interaction (Class III).^[^ ^22^, ^23^, ^24^, ^25^, ^26^, ^27^, ^28^, ^29^ ^]^ Class II mixed‐valence systems exhibiting valence tautomerism have attracted significant attention from early studies to recent research because their valence states can be reversibly modulated through thermal or optical pathways, resulting in changes in color, magnetism, and structure. Intervalence charge transfer (IVCT) absorption is a representative phenomenon of mixed‐valence systems,^[^ ^30^, ^31^, ^32^ ^]^ typically observed through temperature‐dependent spectroscopy and spectroelectrochemistry.^[^ ^33^, ^34^, ^35^, ^36^, ^37^ ^]^

Prussian blue analogs, which are representative cyanido‐bridged metallic assemblies, are known as Class II mixed‐valence materials. Some cyanido‐bridged metal assemblies exhibit phase transitions due to CT between the metal ions. The CT phase transitions can be controlled with external stimuli of heat, light, and electric current.^[^ ^38^, ^39^, ^40^, ^41^, ^42^, ^43^, ^44^, ^45^, ^46^, ^47^ ^]^ Cyanido‐bridged cobalt‐tungstate (CoW) assemblies, including redox‐active transition metals of Co (Co^II/III^) and octacyanidotungstate (W^IV/V^), show phase transitions based on the CT‐induced spin transition (CTIST) between the high temperature (HT) high spin (HS) Co^II^_HS_–W^V^ state and the low temperature (LT) low spin (LS) Co^III^_LS_–W^IV^ state.^[^ ^48^, ^49^, ^50^, ^51^ ^]^ The phase transitions are accompanied by a thermal hysteresis loop derived from the strong cooperativity of cyanido‐bridged networks with the characteristic absorption due to a heterometallic IVCT between Co and W in the visible–near‐infrared (NIR) region.^[^ ^52^, ^53^, ^54^ ^]^ In the cyanido‐bridged CoW assemblies, the organic ligands coordinated to the Co sites influence the charge‐transfer phase transitions through intermolecular cooperativity, and the redox activity of Co sites is governed by the ligand fields. We focused on pyrazine ligands bridging redox‐active metal sites, which give rise to Class II mixed‐valence IVCT bands in the NIR region, as observed in Fe and Ru complexes.^[^ ^55^, ^56^, ^57^, ^58^, ^59^ ^]^ In the present work, we synthesized a new photomagnet with a 3D cyanido‐bridged CoW network, Co_8_[W(CN)_8_]_5_Cl·(pyrazine)_11_·21H_2_O (**1**), showing the multistep thermal phase transition and the photoinduced phase transition associated with the CTIST between cyanido‐bridged Co and W and the IVCT between pyrazine‐bridged Co^II^ and Co^III^ sites. The homometallic Co^II/III^ mixed valency is generated by the heterometallic Co–W CT phase transition. Although many mixed‐valence systems based on Ru^II/III^‐ and Fe^II/III^‐pyrazine have been reported,^[^ ^55^, ^56^, ^57^, ^58^, ^59^ ^]^ as far as we know, this material is the first example of the [Co^II^–pyrazine–Co^III^] mixed valence system. This article reports the crystal structure and the thermal and photoinduced phase transitions of this material, identified by investigating its magnetic and spectral properties.

## 2 Results and Discussion

### 2.1 Synthesis and Crystal Structure

**Figure 1:**
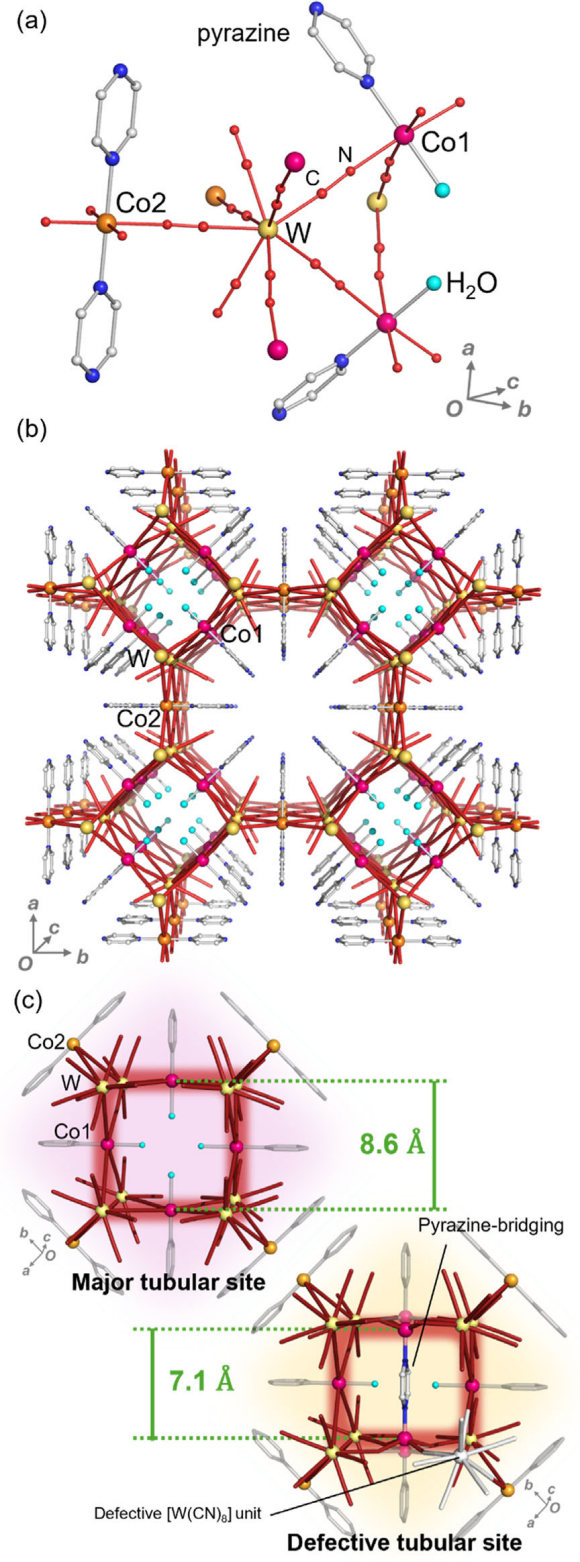
Crystal structure of **1** at room temperature without crystal water molecules. a) Coordination environment of 1. The pink, orange, yellow, and cyan spheres and the red line indicate Co1, Co2, W, O, and b) the cyanido‐bridged network, respectively. The packing structure of 1 is viewed from the *c*‐axis. c) Coordination environment of major and defective tubular sites. The water molecules are substituted to the pyrazine‐bridging between Co1 sites, and one of the [W(CN)_8_]^3−^ sites coordinated to pyrazine‐bridging Co is defective.

A red crystalline powder of **1** was prepared by the slow diffusion method, in which an aqueous solution of CoCl_2_·6H_2_O, pyrazine, and CsCl was added to an aqueous solution of Cs_3_[W(CN)_8_]·2H_2_O and CsCl at room temperature and left to stand for three weeks. Elemental analyses by inductively coupled plasma mass spectrometry for metal ions; the standard method for C, H, N, and Cl; thermogravimetry measurements revealed that the composition of the present compound is Co_8_[W(CN)_8_]_5_Cl·(pyrazine)_11_·21H_2_O. Since the obtained single crystal is tiny, we employed the microcrystal electron diffraction (MicroED) to reveal the crystal system, space group, and the cyanido‐bridged network. The MicroED measurement conducted at room temperature under vacuum indicates that the present compound has a tetragonal structure with the *P*4/*mmm* space group (**Figure** **1** and S4–S6, Table S1–S3, Supporting Information).^[^ ^60^ ^]^ The asymmetric unit of **1** is composed of a [W^V^(CN)_8_]^3−^ anion, a [Co(pyrazine)(H_2_O)(NC)_4_]^2+^ cation (Co1), and one half of a [Co(pyrazine)_2_(NC)_4_]^2+^ cation (Co2). The coordination geometry of the W site is close to a bicapped trigonal prism (Table S2, Supporting Information). The six cyanide groups of [W(CN)_8_]^3−^ are occupied at four Co1 sites and two Co2 sites, and the others remain terminal. The Co1 and Co2 sites adopt a pseudooctahedral six‐coordinated geometry, which is coordinated by four N atoms of cyanide ligands of [W(CN)_8_]^3−^ in equatorial positions for both Co sites. In the axial positions, Co1 is coordinated by one N atom from a pyrazine ligand and one O atom from a water molecule, whereas Co2 is coordinated by two N atoms from two pyrazine ligands. The cyanido‐bridged Co1‐W network forms a rectangular tubular structure along the *c*‐axis, and the tubes are bound by the Co2 sites, resulting in a 3D network with pores (Figure 1b), where the vacant space in the unit cell should be occupied by water molecules. However, such water molecules in the pores could not be assigned in the MicroED analysis because under the vacuum condition, they are released from the pores, leading to severe disorders and uncertainty of their positions. We also conducted a powder X‐ray diffraction (PXRD) measurement at room temperature without vacuum, and the Rietveld analysis indicates the same crystal structure with a larger cell volume than that of the MicroED analysis. In the Reitveld analysis, we could not assign the water molecules in the pores, but such a cell expansion at the ambient pressure suggests inclusion of the water molecules in the pores, which have enough volume of 320 Å. Overall, the crystal structures determined by MicroED and PXRD analyses are considered to be those without and with a water molecule in the pores, respectively. Moreover, the MicroED measurement detected the disorder of the Co1 site and electron density between the Co1 sites facing each other in the tubular structure, and the PXRD measurement revealed that there is a major tubular site with a distance between facing Co1 sites of 8.6 Å and a minor site with a distance of 7.1 Å (Figure 1c). Based on the elemental analyses and crystal structure, ≈1/16 of the [W(CN)_8_]^3−^ sites are defective. Consequently, the Co1 sites in the defective structure should be coordinated by the pyrazine ligand, water molecules, and Cl ions. The Co1‐Co1 distance in the defective structure is consistent with the pyrazine bridging, as supported by the residual electron density assigned to the pyrazine ligand in the tubular defective structure in the MicroED analysis results shown in Figure 1c. It should be noted that the positions of the water molecules and Cl ions at the defective [W(CN)_8_]^3−^ sites could not be resolved in both MicroED and PXRD analyses, and that their existences are owed to the elemental analysis.

### 2.2 Multistep Thermal Phase Transition

**Figure 2:**
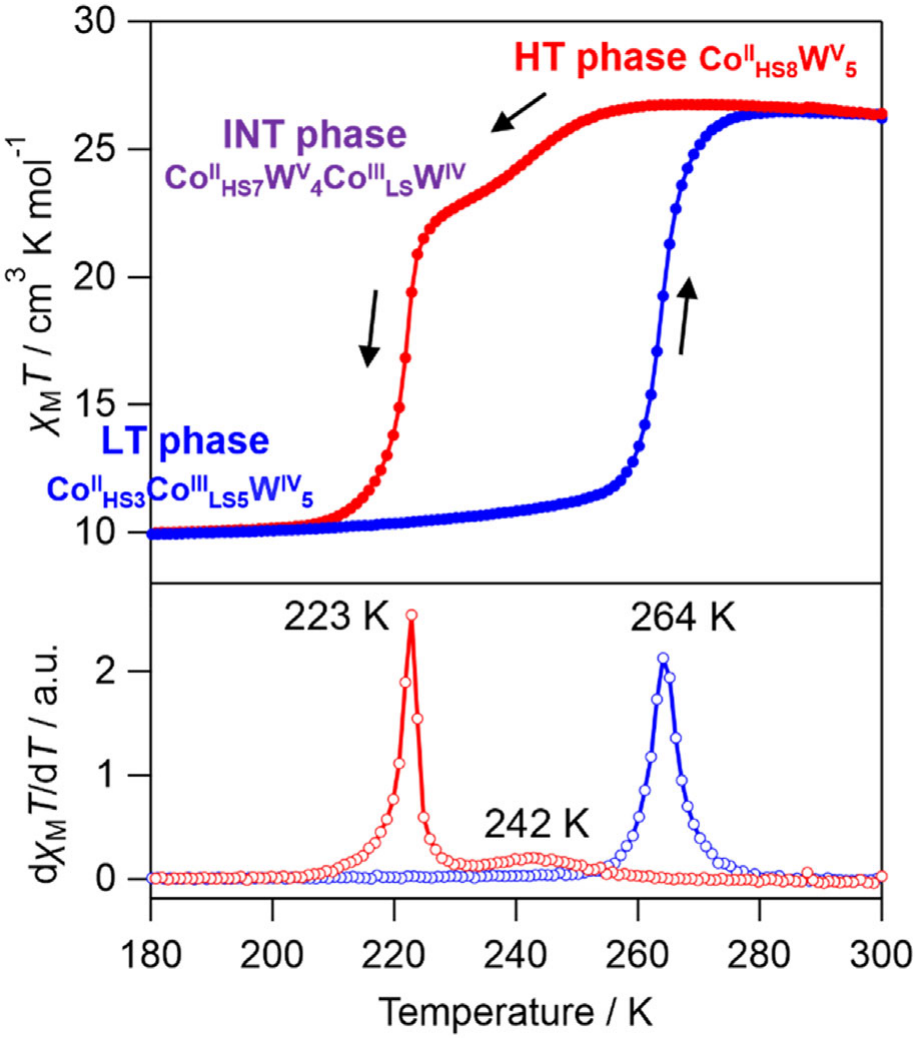
Temperature dependence of *χ*_M_ *T* of **1** (top) upon cooling (red) and heating (blue), and temperature dependence of the first derivative of *χ*_M_ *T* (d*χ*_M_ *T*/d*T*, bottom). The schematic illustration represents the electronic states of the Co sites in the LT, INT, and HT phases.

The temperature (*T*) dependence of the molar magnetic susceptibility (*χ*_M_) of **1** shows drastic changes with an asymmetric thermal hysteresis loop (**Figure** **2** and S7, Supporting Information). The *χ*_M_ *T* value is 28.6 cm^3^ K mol^−1^ at 300 K, which corresponds to the expected value of 28.2 cm^3^ K mol^−1^ for Co^II^_HS8_W^V^_5_ (Co^II^_HS_: *g* = 2.65, *S*_Co_ = 3/2, W^V^: *g*_W_ = 2.00, *S*_W_ = 1/2), denoted as the HT phase. Upon cooling, the *χ*_M_ *T* value remains around 250 K, decreases to 24.8 cm^3^ K mol^−1^ at 232 K with a small step, and drastically decreases to 10.3 cm^3^ K mol^−1^ at 100 K with a second step; these values agree with the expected values of 24.5 cm^3^ K mol^−1^ for Co^III^_LS_W^IV^Co^II^_HS7_W^V^_4_ and 9.9 cm^3^ K mol^−1^ for Co^III^_LS5_W^IV^_5_Co^II^_HS3_ (Co^III^_LS_: *S*_Co_ = 0, W^IV^: *S*_W_ = 0, Co^II^: *g* = 2.65, *S*_Co_ = 3/2). The phases after the first and second steps are named the intermediate (INT) phase and the LT phase, respectively. Upon heating, the *χ*_M_ *T* value of the LT phase is maintained until 250 K, and it returns to the initial value of the HT phase at ≈260 K in one step. The multistep phase transition behavior is retained for at least five cycles, and the shift of the hysteresis loop is likely caused by the desorption of water molecules from the pores of the crystal structure (Figure S7, Supporting Information). Considering the crystal structure, upon cooling from the HT phase, the phase transition to the INT phase originates from a CTIST occurring at the defective tubular site, which contains three Co1‐W pairs due to the absence of one W site. The subsequent transition to the LT phase arises from a CTIST at the other three intact tubular sites. Furthermore, the variable‐temperature PXRD measurements reveal that the LT phase at 150 K exhibits a PXRD pattern similar to that of the HT phase, but with a shift to a higher 2*θ* angle and without peak splitting (Figure S9, Supporting Information). This result suggests that the LT phase retains the same crystal system as the HT phase, accompanied by the lattice contraction. Comparison of the magnetic properties and ratio of Co sites in the asymmetric unit indicates that the Co1 site is responsible for the phase transition. The PXRD pattern at 240 K appears broader than those of both the HT and LT phases, likely due to a mixture of the two phases. The field‐cooled magnetization (FCM) curve of the LT phase shows no clear critical temperature, indicating paramagnetic behavior (Figure S8, Supporting Information).

### 2.3 Variable‐Temperature IR and UV–vis‐NIR Spectra

**Figure 3:**
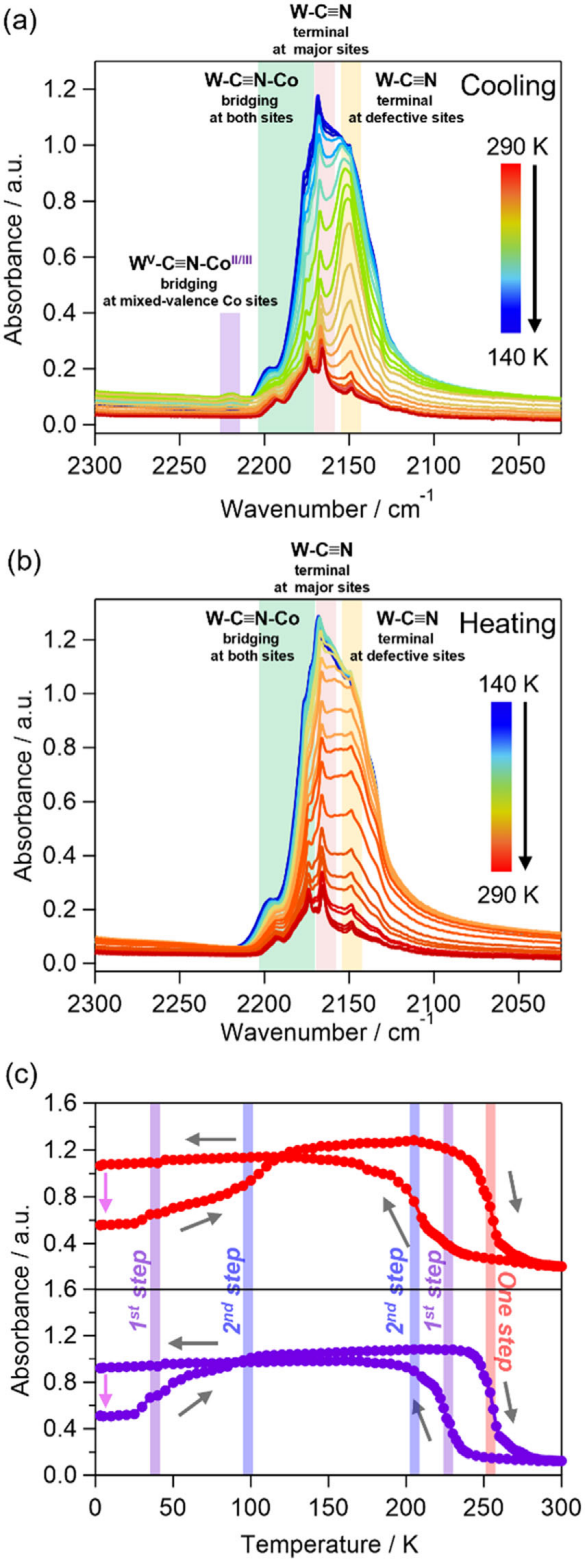
Variable‐temperature IR spectra of cyanide stretching modes on a) cooling and b) heating. c) Temperature trace of the peaks at 2160 cm^−1^ (red) and 2148 cm^−1^ (purple). Purple and blue lines represent the transition temperatures of first and second steps on cooling and on heating to the initial LT phase around 150 K after photoirradiation at 4 K (785 nm, 220 mW cm^−2^, 15 min.), and an orange line indicates a transition temperature on heating to the initial HT phase. Gray and pink arrows indicate the directions of temperature sweeping and the changes induced by the photoirradiation.

The CT phase transition between the HT, INT, and LT phases was also investigated by variable‐temperature IR and UV–vis‐NIR spectroscopy. The IR spectrum of **1** at room temperature shows the pyrazine ring bending mode in the range from 1000 cm^−1^ to 1500 cm^−1^, water bending mode at ≈1600 cm^−1^, cyanide stretching modes (C≡N) at 2100–2200 cm^−1^, and C–H stretching mode of pyrazine at ≈3000 cm^−1^ (Figure S2, Supporting Information). In the cyanide stretching modes, the peaks at 2148 cm^−1^ and 2166 cm^−1^ can be assigned to the terminal W^V^–C≡N modes at the defective and major sites, respectively, whereas the peaks at 2173 cm^−1^ and 2192 cm^−1^ can be attributed to bridging W^V^–C≡N–Co^II^_HS_ modes at both sites.^[^ ^61^, ^62^, ^63^ ^]^ When the temperature decreases to 230 K, the peak at 2148 cm^−1^ significantly increases and slightly shifts to 2150 cm^−1^, and the other peaks gradually grow (**Figure** **3**). In addition, a new weak peak appears at 2220 cm^−1^. On further cooling to 210 K, the peaks at 2166 cm^−1^, 2173 cm^−1^, and 2192 cm^−1^ increase and shift to 2168 cm^−1^, 2177 cm^−1^, and 2198 cm^−1^, respectively. On the contrary, the weak peak at 2220 cm^−1^ decreases and disappears from 230 K to 210 K. The increases in the peak intensities correspond to the CT phase transitions producing the W^IV^–C≡N and W^IV^–C≡N–Co^III^_LS_ modes. The different behavior of the peak at 2220 cm^−1^ suggests the bridging cyanido stretching mode in the defective site with the mixed‐valence Co sites (W^V^–C≡N–Co^II/III^) as supported by the UV–vis results below. Upon heating, the peaks assigned to the LT phase are retained up to 250 K, and the initial state is recovered in one step, similar to the magnetic susceptibility (Figure 3b,c).

The UV–vis‐NIR spectra of the HT phase at room temperature exhibit an absorption peak at 480 nm, which can be assigned to the heterometallic IVCT band from Co^II^ to W^V^ (Figure S3 and S11a, Supporting Information).^[^ ^54^, ^64^, ^65^ ^]^ Upon cooling, the peak intensity at 480 nm decreases at 240 K, and peaks at 980 nm and 2300 nm appear, which can be attributed to the heterometallic IVCT from W^IV^ to Co^III^ and the homometallic IVCT of [Co^III^–pyrazine–Co^II^] originating from the Class II mixed‐valence of Co sites in the defective tubular site, respectively.^[^ ^52^, ^53^, ^54^, ^55^, ^56^, ^57^, ^66^ ^]^ When the temperature is lowered further, the peak at 480 nm decreases more, and a peak at 420 nm becomes apparent, which corresponds to the d–d transition of Co^III^_LS_.^[^ ^67^, ^68^ ^]^ In addition, the peak at 980 nm is merged by the appearance of the peak at 680 nm, attributed to the IVCT from W^IV^ to Co^III^ in the major tubular sites, whereas the peak at 2300 nm disappears because of the transformation from the mixed valence of Co^III^–pyrazine–Co^II^ to Co^III^–pyrazine–Co^III^ in the defective tubular site. Upon heating, the peak at 680 nm decreases, and the peak at 480 nm recovers around 260 K without showing the IVCT band of mixed‐valence Co^II/III^ at 2300 nm (Figure S11b, Supporting Information). The different behaviors upon heating and cooling suggest that the mechanisms of the phase transitions are not the same in the two processes.^[^ ^69^ ^]^ In the thermal phase transitions, the transition temperature depends on the crystal structure, its HT phase stability, and structural cooperativity.^[^ ^70^ ^]^ In the present case, comparison of the major and defective sites indicates that the defective site possesses a more flexible and weaker cyanido‐bridged network due to the presence of defective [W(CN)_8_]^3−^ sites, resulting in lower structural cooperativity and higher HT phase stability. Consequently, the defective site is expected to exhibit a higher transition temperature and a smaller thermal hysteresis loop than the major site. Upon cooling, since the phase transition of the defective site occurs first at the molecular level, we observed a partial IVCT absorption band in the spectroscopic measurements. In contrast, upon heating, the proximity of both transition temperatures due to the smaller hysteresis of the defective site, and the domain of the HT phase generated by the transition of the major site simultaneously induces the transition of the defective site. As a result, neither an IVCT absorption band nor a stepwise transition was observed during heating. Overall, the existence of the defective tubular site with the pyrazine bridging affecting the thermal phase transition on the cooling process is supported not only by the MicroED and magnetic study results, but also by the spectroscopic analysis results. Homometallic IVCT absorption bands are typically generated by redox reactions induced through chemical or electrochemical treatments or by temperature‐induced electron transfer between metal ions and ligands.^[^ ^36^, ^57^, ^65^ ^]^ Interestingly, however, in the present material, the homometallic IVCT absorption band associated with the [Co^III^–pyrazine–Co^II^] unit appears due to the phase transitions driven by the CTIST from the Co^II^_HS_‐W^V^ state to the Co^III^_LS_‐W^IV^ state.

### 2.4 Photoinduced Phase Transition

**Figure 4:**
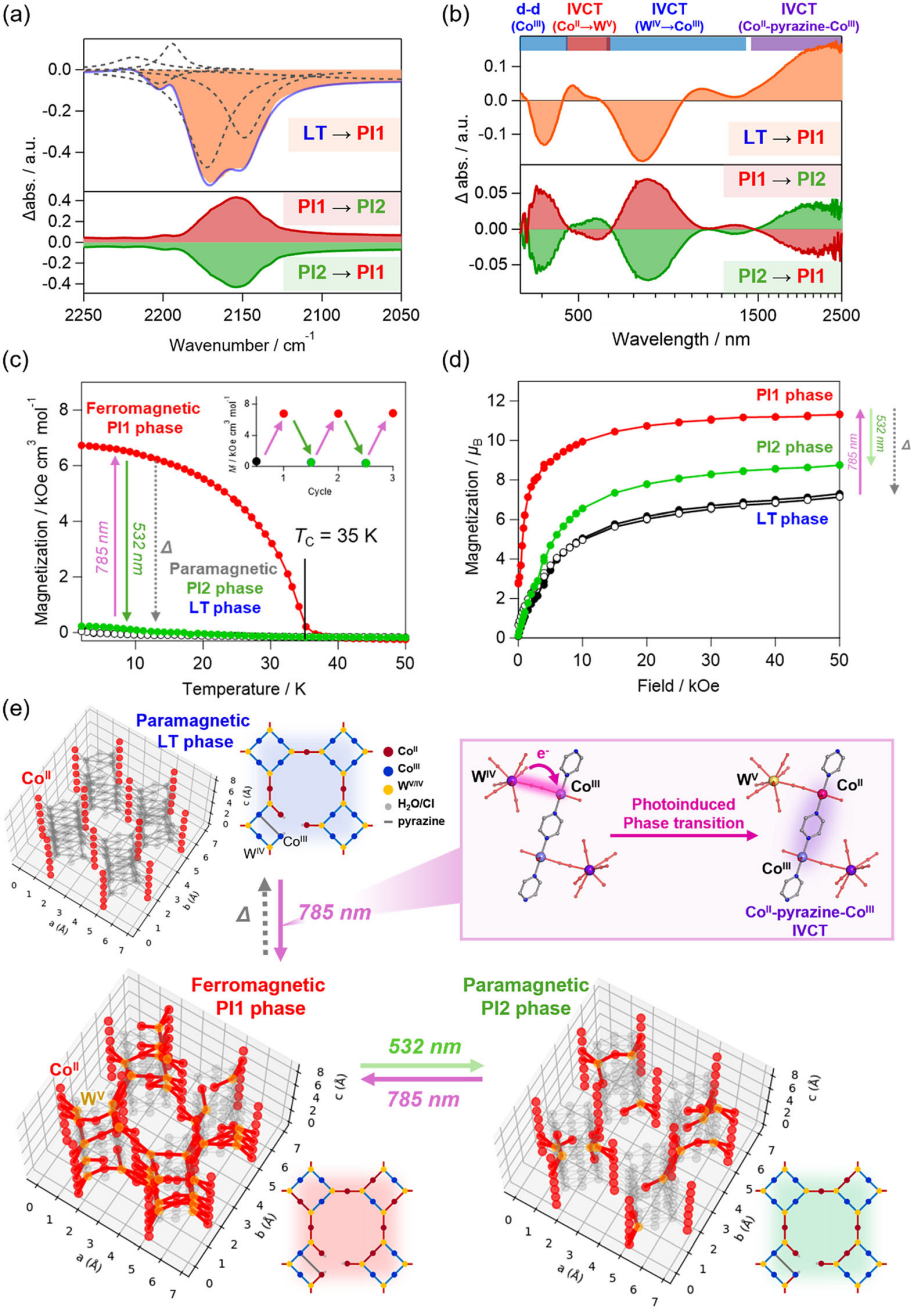
a) Photoinduced IR spectral difference in the range of the cyanide stretching mode. The orange, red, and green areas and blue and gray lines show the spectral changes obtained by subtracting the LT phase from the PI1 phase, PI1 phase from the PI2 phase, and PI2 phase from the PI1 phase and the fitting results for the difference between the LT and PI1 phases and fitting components, respectively. b) Photoinduced UV–vis‐NIR spectral difference. The orange, red, and green areas show the spectral changes obtained by subtracting the LT phase from the PI1 phase, PI1 phase from the PI2 phase, and PI2 phase from the PI1 phase, respectively. The PI1 and PI2 phases were generated by photoirradiation to the LT phase with 785 nm (220 mW cm^−2^, 10 min) and to the PI1 phase with 532 nm (260 mW cm^−2^, 10 min). c) FCM curves under an external field of 100 Oe with photoirradiation. d) Initial *M*–*H* curves at 2 K. The black, red, unfilled black, and green circles indicate before and after photoirradiation (785 nm, 160 mW cm^−2^, 10 min), after thermal annealing at 150 K, and after photoirradiation to the PI1 phase (532 nm, 110 mW cm^−2^, 10 min), respectively. e) Schematic and simulated illustrations of the magnetic networks for the LT, PI1, and PI2 phases.

The photoresponsivity of the LT phase was investigated using IR and UV–vis‐NIR spectroscopy and magnetic measurements. The photoirradiation at 785 nm was chosen to excite the heterometallic IVCT band from W^IV^ to Co^III^_LS_, centered at 680 nm. In the IR spectra, upon photoirradiation of 785 nm at 3 K, the cyanide stretching peaks at 2160 cm^−1^ in the LT phase decrease, indicating that the photoinduced CT occurs at both tubular sites. This state is referred to as the photoinduced 1 (PI1) phase (**Figure** **4**a). The multi‐peak fitting for the difference absorption spectrum between the PI1 and LT phases reveals that it is composed of three negative components coming from the decreases in the W^IV^–C≡N–Co^III^_LS_ and W^IV^–C≡N bands and two positive components from the W–C≡N–Co^II/III^ and W^V^–C≡N–Co^II^_HS_ bands. Remarkably, upon heating of the PI1 phase, the peak at 2148 cm^−1^ returns to the initial LT phase at 50 K as a first step, and the peaks at 2166 cm^−1^ and 2173 cm^−1^ recover to the initial LT phase at 110 K as a second step (Figure S10, Supporting Information). Moreover, we investigated the photoreversibility using 532 nm light exciting the heterometallic IVCT from Co^II^ to W^V^. In the cyanide stretching modes of the PI1 phase, the peak at 2148 cm^−1^ recovers almost completely, and the peaks at 2166 cm^−1^ and 2173 cm^−1^ return partially, producing the PI2 phase. These results suggest that the reverse‐photoinduced phase transition occurs preferentially at the defective tubular sites rather than the major tubular sites. The PI2 phase can revert to the PI1 phase upon photoirradiation at 785 nm, and the two phases are photoreversible, as confirmed over three cycles (Figure S10, Supporting Information).

In the UV–vis‐NIR spectra of the PI1 phase at 3 K with photoirradiation at 785 nm to the LT phase, the peaks at 410 nm and 750 nm, which are assigned to the d–d transition of Co^III^ and IVCT from W^IV^ to Co^III^_LS_, decrease. In addition, the peaks at ≈500 nm, 1000 nm, and 2300 nm, which are attributed to the IVCT from Co^II^ to W^V^, IVCT from W^IV^ to Co^III^ of the mixed‐valence Co^II/III^ sites, and homometallic IVCT of [Co^III^–pyrazine–Co^II^], respectively, increase (Figure 4b).^[^ ^55^, ^56^, ^57^ ^]^ The IVCT band of [Co^III^–pyrazine–Co^II^] is more clearly observed compared to that at 240 K during cooling, indicating that the thermal phase transition upon cooling occurs more selectively at the defective sites, leading to a predominance of [Co^III^–pyrazine–Co^III^] sites, which do not exhibit the IVCT band. Upon 532 nm photoirradiation of the PI1 phase, the peaks at 500 nm and 2300 nm decrease, whereas those at 410 nm and 750 nm increase. We also confirmed the photoreversibility between the PI1 and PI2 phases, as evidenced by the completely symmetric differential absorption spectra observed upon photoirradiation between the two phases (Figure 4b). This photoreversible behavior was further validated by repeating the measurements three times (Figure S11, Supporting Information).

The photomagnetism of **1** was evaluated by magnetic measurements before and after photoirradiation. As mentioned above, the LT phase shows paramagnetism. The 785 nm photoirradiation of the LT phase at 3 K under an external magnetic field of 100 Oe causes an increase in magnetization. The FCM curve of the PI1 phase shows a Curie temperature of 35 K (Figure 4c). In the magnetization (*M*) versus magnetic field (*H*) plots at 2 K, the saturation magnetization values before and after photoirradiation are 6.8 *μ*_B_ and 11.4 *μ*_B_ at 50 kOe, respectively, and the magnetic hysteresis loop after the photoirradiation has a small coercive field (*H*_C_) of 500 Oe (Figure 4d and S12, Supporting Information). The saturation magnetization values of the LT and PI1 phases are close to those of 6.5 *μ*_B_ for Co^III^_LS5_W^IV^_5_Co^II^_HS3_ and 11.2 *μ*_B_ for Co^III^_LS3.5_W^IV^_3.5_Co^II^_HS4.5_W^V^_1.5_, assuming ferromagnetic interactions between Co^II^ (Kramers doublet: *g* = 13/3, *S*_Co_ = 1/2) and W^V^ (*g*_W_ = 2.00, *S*_W_ = 1/2). Although the PI1 phase contains a considerable amount of diamagnetic Co^III^ and W^IV^ species, its Curie temperature is relatively high compared to those of previously reported cyanido‐bridged CoW assemblies.^[^ ^49^, ^50^, ^51^, ^52^, ^53^ ^]^ This difference exists because six of the eight cyanide ligands in the octacyanidotungstate coordinate to the Co sites, enabling multiple magnetic couplings within the 3D coordination network. The magnetization value of the PI1 phase returns to that of the initial LT phase by thermal annealing at 150 K. The photothermal reversibility between the LT and PI1 phases was confirmed three times (Figure S12, Supporting Information). Upon 532 nm irradiation of the PI1 phase, the magnetization significantly decreases, resulting in paramagnetism (Figure 4c). The *M*–*H* plot of the PI2 phase exhibits a saturated magnetization of 8.6 *μ*_B_, which is estimated to correspond to the calculated value of 8.4 *μ*_B_ for Co^III^_LS4.4_W^IV^_4.4_Co^II^_HS3.6_W^V^_0.6_, indicating the partial transition from the Co^II^_HS_‐W^V^ state to the Co^III^_LS_‐W^IV^ state (Figure 4d). The photoreversibility between the PI1 and PI2 phases was reproduced by three cycles (Figure 4c inset). The PI2 phase also returns to the initial LT phase by thermal annealing at 150 K. Furthermore, comparison of the *χ*_M_ *T*–*T* plots during the thermal annealing processes of the PI1 and PI2 phases reveals multiple relaxation steps at 50 K and 110 K, which are also supported by the IR spectral data (Figure S12, Supporting Information).

To investigate the magnetic ordering further, the magnetic networks in the LS, PI1, and PI2 phases were examined. In this analysis, the cyanido‐bridged connections between the Co^II^ and W^V^ sites were treated as magnetically coupled bonds (Figure 4e, red lines), whereas those between the Co^III^ and W^IV^ sites were considered as magnetically disconnected (Figure 4e, gray lines). Additionally, the Co^II^–W^V^ and Co^III^–W^IV^ ratios determined from the magnetic measurements described above were used. Based on these calculations, in the PI1 phase, 24.9% (±1.3%) of the cyanido bridges are magnetically coupled, which corresponds to 4.2 cyanide bridges per octacyanidetungstate(V) unit. This value likely represents the lower limit required to maintain the 3D magnetic network. Therefore, as the numbers of Co^II^ and W^V^ spin sites decrease, the PI2 phase, which has 7.4% (±0.8%) magnetically coupled bonds (3.7 cyanide bridges per the remained octacyanidetungstate(V) unit), can no longer retain the magnetically ordered state.

## 3 Conclusion

We prepared a new photomagnetic material based on a cyanido‐bridged Co–W assembly, Co_8_[W(CN)_8_]_5_Cl·(pyrazine)_11_·21H_2_O, which exhibits an IVCT band associated with mixed‐valence Co^II/III^ sites bridged by pyrazine ligands. The present compound features a 3D network structure composed of crystallographically independent Co (Co1 and Co2) and W sites. The Co1 sites, located within the tubular structures, participate in the phase transition driven by heterometallic CT between the Co and W sites, whereas the Co2 sites remain in the HS Co^II^ state. Additionally, the tubular structure contains defects at the [W(CN)_8_] sites, where pyrazine bridges between two Co1 sites ([Co1–pyrazine–Co1]) can form. The presence of these defect sites enables a multistep thermal phase transition and the emergence of homometallic IVCT of the [Co^II^–pyrazine–Co^III^] unit. Interestingly, the homometallic IVCT band at 2300 nm appears transiently and then disappears upon cooling, corresponding to a transformation from [Co^II^–pyrazine–Co^III^] to [Co^III^–pyrazine–Co^III^], as the number of Co^III^ sites increases due to the CT phase transition. Photoirradiation at 785 nm induces a transition from the LT phase to the PI1 phase, which exhibits ferromagnetism with a Curie temperature of 35 K, due to the photoinduced CTIST between the Co and W sites. The PI1 phase also exhibits a homometallic IVCT from the [Co^II^–pyrazine–Co^III^] unit. Furthermore, photoirradiation with 532 nm light induces a transition from the PI1 phase to the paramagnetic PI2 phase. This magnetic change—from ferromagnetic to paramagnetic—is attributed to the significant decrease in the magnetically coupled bonds between the Co and W sites, caused by the increase in diamagnetic Co^III^ and W^IV^ species. The presence of pyrazine bridges between the redox‐active Co^II/III^ sites in the phase transition material enables the formation of both thermally and photoinduced IVCT absorption bands in the NIR region through the CT phase transitions. This study reveals a notable modulation of electronic states in the CT photomagnet achieved through the homo‐ and heterometallic CT processes. Such a material holds potential for additional photoresponsivity arising from excitation of the near‐IR IVCT band, as well as for thermal and electric switching of physical properties via valence tautomerism, offering new functionalities for phase transition materials and photomagnets.

## Supporting Information

Supporting Information is available from the Wiley Online Library or from the author.

## Conflict of Interest

The authors declare no conflict of interest.

## Supporting information

Supplementary Material

## Data Availability

The data that support the findings of this study are available from the corresponding author upon reasonable request.

## References

[1] T.‐T. Lu , Y.‐M. Wang , C.‐H. Hung , S.‐J. Chiou , W.‐F. Liaw , Inorg. Chem. 2018, 57, 12425. 30247022 10.1021/acs.inorgchem.8b01818

[2] S. Koshihara , T. Ishikawa , Y. Okimoto , K. Onda , R. Fukaya , M. Hada , Y. Hayashi , S. Ishihara , T. Luty , Phys. Rep. 2022, 942, 1.

[3] R. Bertoni , M. Lorenc , H. Cailleau , A. Tissot , J. Laisney , M.‐L. Boillot , L. Stoleriu , A. Stancu , C. Enachescu , E. Collet , Nat. Mater. 2016, 15, 606. 27019383 10.1038/nmat4606

[4] M. A. C. Stuart , W. T. S. Huck , J. Genzer , M. Müller , C. Ober , M. Stamm , G. B. Sukhorukov , I. Szleifer , V. V. Tsukruk , M. Urban , F. Winnik , S. Zauscher , I. Luzinov , S. Minko , Nat. Mater. 2010, 9, 101. 20094081 10.1038/nmat2614

[5] A. G. Majouga , M. I. Zvereva , M. P. Rubtsova , D. A. Skvortsov , A. V. Mironov , D. M. Azhibek , O. O. Krasnovskaya , V. M. Gerasimov , A. V. Udina , N. I. Vorozhtsov , E. K. Beloglazkina , L. Agron , L. V. Mikhina , A. V. Tretyakova , N. V. Zyk , N. S. Zefirov , A. V. Kabanov , O. A. Dontsova , J. Med. Chem. 2014, 57, 6252. 24950478 10.1021/jm500154f

[6] P. Zhang , P. J. Sadler , Eur. J. Inorg. Chem. 2017, 2017, 1541.

[7] G. Molnár , S. Rat , L. Salmon , W. Nicolazzi , A. Bousseksou , Adv. Mater. 2018, 30, 1703862. 10.1002/adma.201703862 29171924

[8] O. Kahn , C. J. Martinez , Science 1998, 279, 44.

[9] S. Ohkoshi , S. Takano , K. Imoto , M. Yoshikiyo , A. Namai , H. Tokoro , Nat. Photon. 2014, 8, 65.

[10] T. Mallah , S. Thiébaut , M. Verdaguer , P. Veillet , Science 1993, 262, 1554. 17829385 10.1126/science.262.5139.1554

[11] E. Collet , M.‐H. Lemée‐Cailleau , M. Buron‐Le Cointe , H. Cailleau , M. Wulff , T. Luty , S.‐Y. Koshihara , M. Meyer , L. Toupet , P. Rabiller , S. Techert , Science 2003, 300, 612. 12714737 10.1126/science.1082001

[12] G. M. Mínguez Espallargas , E. Coronado , Chem. Soc. Rev. 2018, 47, 533. 29112210 10.1039/c7cs00653e

[13] A. Ueda , K. Kishimoto , T. Isono , S. Yamada , H. Kamo , K. Kobayashi , R. Kumai , Y. Murakami , J. Gouchi , Y. Uwatoko , Y. Nishio , H. Mori , RSC Adv. 2019, 9, 18353. 35515234 10.1039/c9ra02833a PMC9064737

[14] M. Shivanna , Q.‐Y. Yang , A. Bajpai , E. Patyk‐Kazmierczak , M. J. Zaworotko , Nat. Commun. 2018, 9, 3080. 30082776 10.1038/s41467-018-05503-y PMC6079025

[15] M. Sutradhar , M. V. Kirillova , M. F. C. Guedes da Silva , Inorg. Chem. 2012, 51, 11229. 23098259 10.1021/ic3017062

[16] M. A. Halcrow , Coord. Chem. Rev. 2009, 253, 2493.

[17] J. S. Miller , Chem. Soc. Rev. 2011, 40, 3266.21479292

[18] Y. Sagara , T. Mutai , I. Yoshikawa , K. Araki , J. Am. Chem. Soc. 2007, 129, 1520. 17249676 10.1021/ja0677362

[19] E. S. Koumousi , I. R. Jeon , Q. Gao , P. Dechambenoit , D. N. Woodruff , P. Merzeau , L. Buisson , X. Jia , D. Li , F. Volatron , C. Mathonière , R. Clérac , J. Am. Chem. Soc. 2014, 136, 15461. 25298164 10.1021/ja508094h

[20] A. Bakandritsos , R. G. Kadam , P. Kumar , G. Zoppellaro , M. Medved , J. Tuček , T. Montini , O. Tomanec , P. Andrýsková , B. Drahoš , R. S. Varma , M. Otyepka , M. B. Gawande , P. Fornasiero , R. Zbořil , Adv. Mater. 2019, 31, e1900323. 30811705 10.1002/adma.201900323

[21] C. T. Kelly , S. Dunne , I. A. Kühne , A. Barker , K. Esien , S. Felton , H. Müller‐Bunz , Y. Ortin , G. G. Morgan , Angew. Chem. Int. Ed. 2023, 62, e202217388. 10.1002/anie.202217388 36794891

[22] E. Göransson , R. Emanuelsson , K. Jorner , T. F. Markle , L. Hammarström , H. Ottosson , Chem. Sci. 2013, 4, 3522.

[23] D. M. D’Alessandro , F. R. Keene , Chem. Soc. Rev. 2006, 35, 424. 16636726 10.1039/b514590m

[24] S. Zhang , D. K. Panda , A. Yadav , W. Zhou , S. Saha , Chem. Sci. 2021, 12, 13379. 34777756 10.1039/d1sc04338b PMC8528024

[25] M. B. Robin , P. Day , Adv. Inorg. Chem. Radiochem. 1968, 10, 247.

[26] T. Matsumoto , G. N. Newton , T. Shiga , S. Hayami , Y. Matsui , H. Okamoto , R. Kumai , Y. Murakami , H. Oshio , Nat. Commun. 2014, 5, 3865. 24832549 10.1038/ncomms4865

[27] K. D. Demadis , C. M. Hartshorn , T. J. Meyer , Chem. Rev. 2001, 101, 2655. 11749392 10.1021/cr990413m

[28] D. A. Shultz , Magnetism: Molecules To Materials II, John Wiley & Sons, Limited 2001, pp. 281–306.

[29] T. Tezgerevska , K. G. Alley , C. Boskovic , Coord. Chem. Rev. 2014, 268, 23.

[30] F. W. Vance , L. Karki , J. K. Reigle , J. T. Hupp , M. A. Ratner , J. Phys. Chem. A 1998, 102, 8320.

[31] D. M. Adams , L. Noodleman , D. N. Hendrickson , Inorg. Chem. 1997, 36, 3966. 10.1021/ic9514307 11669688

[32] D. L. Sun , S. V. Rosokha , S. V. Lindeman , J. K. Kochi , J. Am. Chem. Soc. 2003, 125, 15950. 14677987 10.1021/ja037867s

[33] D. M. D’Alessandro , F. R. Keene , Chem. Rev. 2006, 106, 2270. 16771450 10.1021/cr050010o

[34] M. J. Powers , T. J. Meyer , J. Am. Chem. Soc. 1978, 100, 4393.

[35] E. R. Kearns , B. Chan , H. J. Windsor , W. Lewis , D. M. D’Alessandro , Mater. Adv. 2024, 5, 1588.

[36] C. Carbonera , A. Dei , J.‐F. Létard , C. Sangregorio , L. Sorace , Angew. Chem. Int. Ed. 2004, 43, 3136. 10.1002/anie.200453944 15199559

[37] I. Ramírez‐Wierzbicki , A. Cotic , A. Cadranel , Chemphyschem 2022, 23, e202200384. 35785464 10.1002/cphc.202200384 PMC9805035

[38] D. Pinkowicz , M. Rams , M. Mišek , K. V. Kamenev , H. Tomkowiak , A. Katrusiak , B. Sieklucka , J. Am. Chem. Soc. 2015, 137, 8795. 26098129 10.1021/jacs.5b04303

[39] T. S. Venkatakrishnan , S. Sahoo , N. Bréfuel , C. Duhayon , C. Paulsen , A.‐L. Barra , S. Ramasesha , J.‐P. Sutter , J. Am. Chem. Soc. 2010, 132, 6047. 20380425 10.1021/ja9089389

[40] S. Ohkoshi , H. Tokoro , Acc. Chem. Res. 2012, 45, 1749. 22869535 10.1021/ar300068k

[41] S. Ohkoshi , H. Tokoro , T. Hozumi , Y. Zhang , K. Hashimoto , C. Mathoniere , I. Bord , G. Rombaut , M. Verelst , C. C. D. Moulin , F. Villain , J. Am. Chem. Soc. 2006, 128, 270. 16390157 10.1021/ja0559092

[42] S. Chorazy , J. Stanek , W. Nogas , A. Majcher , M. Rams , M. Kozieł , E. Juszyńska‐Gałązka , K. Nakabayashi , S. Ohkoshi , B. Sieklucka , R. Podgajny , J. Am. Chem. Soc. 2016, 138, 1635. 26761594 10.1021/jacs.5b11924

[43] S. Ohkoshi , K. Nakagawa , M. Yoshikiyo , A. Namai , K. Imoto , Y. Nagane , F. Jia , O. Stefanczyk , H. Tokoro , J. Wang , T. Sugahara , K. Chiba , K. Motodohi , K. Isogai , K. Nishioka , T. Momiki , R. Hatano , Nat. Commun. 2023, 14, 8466. 38151489 10.1038/s41467-023-44350-4 PMC10752886

[44] O. N. Risset , P. A. Quintero , T. V. Brinzari , M. J. Andrus , M. W. Lufaso , M. W. Meisel , D. R. Talham , J. Am. Chem. Soc. 2014, 136, 15660. 25286151 10.1021/ja5084283

[45] S. Chorazy , J. J. Zakrzewski , M. Magott , T. Korzeniak , B. Nowicka , D. Pinkowicz , R. Podgajny , B. Sieklucka , Chem. Soc. Rev. 2020, 49, 5945. 10.1039/d0cs00067a 32685956

[46] N. Yanai , W. Kaneko , K. Yoneda , M. Ohba , S. Kitagawa , J. Am. Chem. Soc. 2007, 129, 3496. 17341085 10.1021/ja069166b

[47] S. M. Holmes , G. S. Girolami , J. Am. Chem. Soc. 1999, 121, 5593.

[48] T. Yoshida , K. Nakabayashi , H. Tokoro , M. Yoshikiyo , A. Namai , K. Imoto , K. Chiba , S. Ohkoshi , Chem. Sci. 2020, 11, 8989. 34123153 10.1039/d0sc02605k PMC8163449

[49] K. Nakamura , K. Nakabayashi , S. Kobayashi , S. Ohkoshi , Inorg. Chem. Front. 2024, 11, 2752.

[50] K. Nakamura , L. Guérin , G. Privault , K. Nakabayashi , M. Hervé , E. Collet , S. Ohkoshi , Nat. Commun. 2025, 16, 5012. 40480987 10.1038/s41467-025-60401-4 PMC12144094

[51] Y. Arimoto , S. Ohkoshi , Z. J. Zhong , H. Seino , Y. Mizobe , K. Hashimoto , J. Am. Chem. Soc. 2003, 125, 9240. 12889922 10.1021/ja030130i

[52] S. Ohkoshi , Y. Hamada , T. Matsuda , Y. Tsunobuchi , H. Tokoro , Chem. Mater. 2008, 20, 3048.

[53] N. Ozaki , H. Tokoro , Y. Hamada , A. Namai , T. Matsuda , S. Kaneko , S. Ohkoshi , Adv. Funct. Mater. 2012, 22, 2089.

[54] K. Nakamura , K. Nakabayashi , K. Imoto , S. Ohkoshi , Inorg. Chem. Front. 2023, 10, 850.

[55] W. Kaim , Coord. Chem. Rev. 2011, 255, 2503.

[56] C. Creutz , P. Kroger , T. Matsubara , T. L. Netzel , N. Sutin , J. Am. Chem. Soc. 1979, 101, 5442.

[57] T. Ito , T. Hamaguchi , H. Nagino , T. Yamaguchi , H. Kido , I. S. Zavarine , T. Richmond , J. Washington , C. P. Kubiak , J. Am. Chem. Soc. 1999, 121, 4625.

[58] F. Felix , A. Ludi , Inorg. Chem. 1978, 17, 1782.

[59] M. Ketterle , W. Kaim , J. Fiedler , Chem. Commun. 1998, 1701.

[60] Deposition Numbers CCDC2450475 and 2475679 for 1 Contain the Supplementary Crystallographic Data for This Paper. These Data Are Provided Free of Charge by the Joint Cambridge Crystallographic Data Center.

[61] B. Sieklucka , R. Podgajny , P. Przychodzén , T. Korzeniak , Coord. Chem. Rev. 2005, 249, 2203.

[62] P. M. Kiernan , W. P. Griffith , J. Chem. Soc. Dalton Trans. 1975, 23, 2489.

[63] H. Tokoro , N. Maeda , K. Imoto , K. Nakabayashi , K. Chiba , S. Ohkoshi , J. Mater. Chem. C 2021, 9, 10689.

[64] K. Nakabayashi , S. Chorazy , M. Komine , Y. Miyamoto , D. Takahashi , B. Sieklucka , S. Ohkoshi , Cryst. Growth Des. 2017, 17, 4511.

[65] S. Chorazy , K. Nakabayashi , K. Imoto , J. Mlynarski , B. Sieklucka , S. Ohkoshi , J. Am. Chem. Soc. 2012, 134, 16151. 22989141 10.1021/ja307520k

[66] N. G. R. Hearns , J. L. Korčok , M. M. Paquette , K. E. Preuss , Inorg. Chem. 2006, 45, 8817. 17054329 10.1021/ic0608997

[67] A. B. P. Lever , S. A. Rice , Phys. Today 1969, 22, 77.

[68] E. Ochiai , K. M. Long , C. R. Sperati , D. H. Busch , J. Am. Chem. Soc. 1969, 91, 3201.

[69] L. J. Kershaw Cook , R. Kulmaczewski , S. A. Barrett , M. A. Halcrow , Inorg. Chem. Front. 2015, 2, 662. 10.1021/ic502726q 25563430

[70] K. Senthil Kumar , N. Del Giudice , B. Heinrich , L. Douce , M. Ruben , Dalton Trans. 2020, 49, 14258. 33026376 10.1039/d0dt02214d

