# Supplementary material for: Homometallic Intervalence Charge Transfer Band of Co(II/III) Induced by Phase Transitions in a Heterometallic Co–W Charge Transfer Photomagnet
Source: Small Sci. 2025 Nov 11;6(1):e202500469. doi: 10.1002/smsc.202500469 (PMC12825454; doi:10.1002/smsc.202500469)
Supplement: Supplementary file 1 — Supplementary Material [file SMSC-6-e202500469-s001.pdf]

SUPPORTING INFORMATION

Table of Contents

1. Experimental details..S2

2. Thermogravimetric measurement of **1** (**Figure S1**)....................................S5

3. IR, Raman, and UV-vis-NIR spectra of **1** at room temperature (**Figures S2 and S3**).....S6

4. Crystal data from ED and PXRD measurements (**Figures S4-S6 and Tables S1-S3**).....S7-S9

5. Magnetic properties of **1** (**Figures S7 and S8**)....................................S10

6. Variable-temperature PXRD measurements (**Figure S9**)................................S11

7. Variable-temperature and photoinduced IR spectra of **1** (**Figure S10**)................S12

8. Variable-temperature and photoinduced UV-vis-NIR spectra of **1** (**Figure S11**)........S13

9. Photomagnetic properties of **1** (**Figure S12**)..S14

10. References for Supporting Information..S15

1. Experimental details

1a. Materials

Cobalt (II) chloride hexahydrate, pyrazine, and caesium chloride were purchased from FUJIFILM Wako. All reagents were used without further purification. The cyanide precursor of $Cs_3[W(CN)_8]\cdot 2H_2O$ was synthesised as described in the literature.[S1,S2]

1b. Synthesis and characterisation of 1

$Co_8[W(CN)_8]_5Cl\cdot(\text{pyrazine})_{11}\cdot 21H_2O$ (**1**) in the crystalline powder was obtained by reacting 2.0 mL of an aqueous solution of $CoCl_2\cdot 6H_2O$ (0.30 mmol), pyrazine (0.40 mmol), and CsCl (0.75 mmol) with 2.0 mL of an aqueous solution containing $Cs_3[W(CN)_8]\cdot 2H_2O$ (0.20 mmol) and CsCl (0.75 mmol), inserting 10 mL of an aqueous buffer solution with CsCl (7.5 mmol) at room temperature in the dark by a slow diffusion method. After three weeks, red needles of tiny crystalline powder were obtained by filtering and washing with small amounts of water and kept in the air for several days (28.1 mg, yield 20.0%). The composition of **1** was determined by C, H, N, and Cl elemental analyses, inductively coupled plasma mass spectrometry (ICP-MS) for Co and W, and thermogravimetry (TG) measurement (Figure S1). Elemental analyses: Calcd. for $Co_8W_5C_{84}H_{88}N_{62}O_{22}Cl_1$ (1: Mw = 3744.14): Co, 12.6%; W, 24.6%; C, 27.0%; H, 2.4%; N, 23.2%, Cl, 1.0%. Found: Co, 12.5%; W, 24.2%; C, 26.9%; H, 2.1%; N, 23.6%. IR spectra; cyanide stretching vibration: 2120 cm^{-1}, 2133 cm^{-1}, 2148 cm^{-1}, 2165 cm^{-1}, 2169 cm^{-1}, and 2192 cm^{-1}. CH stretching vibrations of pyrazine: 3114 cm^{-1} and 3135 cm^{-1} (Figure S2).[S3] Raman spectrum; cyanide stretching vibrations: 2160 cm^{-1} and 2190 cm^{-1} (Figure S2). UV-vis-NIR spectrum; MM´CT from Co^{II} to W^{V}: 480 nm (Figure S3). The density of **1** was evaluated by using a relative density method in which two kinds of liquids with known densities (1,1,2,2-tetrabromoethane, 2.97 g cm^{-3}; N, N-dimethylacetamide, 0.94 g cm^{-3}) were employed to prepare mixed solutions with different densities. The powder of **1** was put into the mixed solution in a test tube, and after waiting for one week, we confirmed that the powder positions in the solutions sunk, floated, and stayed in the middle. The densities of the mixed solutions were set to 1.90 g cm^{-3} and 1.75 g cm^{-3}. The powder of **1** floated and stayed in the middle in the solution, indicating that the density of **1** almost corresponded to 1.75 g cm^{-3}. Considering the chemical formulas matching the elemental analyses based on the empirical formula of the crystal structure, we have two possibilities for the chemical formula: $Co_{3.200}[W(CN)_8]_2Cl_{0.400}\cdot(\text{pyrazine})_{4.400}\cdot 8.40H_2O$ with excess Co sites (Mw = 1497.67 g mol^{-1}, d = 1.883 g cm^{-3}) or $Co_3[W(CN)_8]_{1.875}Cl_{0.375}\cdot(\text{pyrazine})_{4.125}\cdot 7.88H_2O$ with W site defects (Mw = 1404.05 g mol^{-1}, d = 1.766 g cm^{-3}). Therefore, we concluded that the chemical formula of **1** was $Co_8[W(CN)_8]_5Cl\cdot(\text{pyrazine})_{11}\cdot 21H_2O$[1] with W site defects, which is in agreement with the results of the

microcrystal electron diffraction (MicroED) and spectroscopic measurements.

1c. Crystal structure determination

MicroED experiments were conducted using a XtaLAB Synergy-ED system (Rigaku Co. and JEOL Ltd.) equipped with a HyPix-ED detector optimized for operation in the MicroED experimental setup.[S4] The data set was collected using the selected-area diffraction method at room temperature (293 K) at a wavelength of 0.0251 Å, corresponding to an acceleration voltage of 200 kV. The dose rate of the electron beam illuminating the crystals was set to approximately 0.01 e^{-} $Å^{-2}$ s^{-1}. The sample was gently ground between two slide glasses and loaded onto a C-film Cu grid (JEOL Ltd.) by scooping up a slight amount of the sample powder with the grid. All data collection steps, including searching for quality crystals, diffraction image acquisition, intensity extraction, and primary space group determination, were performed using a CrysAlisPro system for ED (Rigaku Co.). During the test, the crystal was rotated continuously at 0.25° s^{-1}, and diffraction images were extracted every 0.5°. The tilt angle range employed for all measurements was −40° to 40°, for a total of 80°. The analysed data were obtained using a tiny single crystal sample with dimensions of 0.9 μm × 0.8 μm × 0.1 μm (Figure S6). The crystal structure was solved by a direct method using SHELXT[S5] and kinematically refined by the full-matrix least-squares method using SHELXL[S6] on the Olex2 single-crystal structure analysis platform.[S7] All non-hydrogen atoms were refined anisotropically, and the hydrogen atoms were refined using a riding model. The crystal structure obtained from the ED measurement was deposited in the Cambridge Crystallographic Data Centre (CCDC 2475679). Powder X-ray diffraction (PXRD) measurements of **1** were conducted by using a Rigaku Ultima IV diffractometer equipped with Cu Kα radiation by the parallel beam method. The diffraction pattern was recorded using the capillary option with a crystalline powder sample in the diffraction angle range of 5°–70° in steps of 0.01° and an exposure time of 0.02 ° min^{-1}, and the rotation ratio was 120° min^{-1} with the Si standard. Rietveld analysis of **1** was performed using the Rigaku PDXL program. The crystal structure was refined based on the crystal structure obtained from the ED measurements and deposited in the Cambridge Crystallographic Data Centre (CCDC 2450475).

1d. Physical techniques

Elemental analyses were performed using the standard microanalytical method for C, H, N, and Cl and with ICP-MS (Agilent 7700x) for Co and W. The TG measurements were conducted using a Rigaku Thermo plus Evo II TG8120. The magnetic properties were investigated using a superconducting quantum interference device (SQUID) magnetometer (Quantum Design MPMS). The temperature-dependent

magnetic susceptibility was measured under an externally applied magnetic field of 5000 Oe. Pascal's constants corrected the diamagnetic contributions from the samples.

Variable-temperature PXRD measurements were conducted using an Ultima IV system with cryogenic options during the cooling process under a vacuum. The measurements were recorded in the range of 5°–60° in steps of 0.02° and an exposure time of 1° min^{-1} with the Si standard. The temperature was controlled down to 150 K by using a model 331 temperature controller from Lakeshore. The measured sample was prepared by pasting the sample powder with grease (Apiezon L) on the Cu sample holder. The IR spectra at room temperature were measured by using a spectrometer (JASCO FT/IR-4100). The measured samples were prepared by dispersing them in KBr. The UV-vis-NIR spectrum at room temperature was obtained by using a spectrometer (JASCO V-670). The sample was prepared by dispersing in $BaSO_4$. Variable-temperature IR and UV-vis-NIR spectra were measured using a Shimadzu FT-IR8200PC spectrometer and UV-3600 plus spectrometer, respectively, with an Oxford Instruments Microstate-He cryostat and 785 nm and 532 nm continuous wavelength (cw) diode lasers. The sample was prepared by dispersing paraffin oil sandwiched between CaF_2 for IR spectroscopy and quartz plates for UV-vis-NIR spectroscopy. Both measurements with photoirradiation were conducted at 4 K. Photomagnetism measurements were conducted by SQUID using an optical fibre whose edge was connected with the sample and 785 nm and 532 nm cw diode lasers. The sample for the photomagnetic measurements was prepared by dispersing ground powder of **1** in the cut transparent label sticker. The photoirradiation in the photomagnetic measurements was conducted at 3 K.

1e. Calculation

The number of magnetically coupled bonds was estimated based on the crystal structure containing a defective site. In the calculation, each elemental component was classified as either in a spin state (Co^{II}_{HS} or W^{V}) or a non-spin state (Co^{III}_{LS} or W^{IV}). The W and Co2 sites in the LT phase were initially set to the non-spin and spin states, respectively. The Co1 sites at the major site were set to the non-spin state, whereas at the defective site, one-fourth of the Co1 sites were set to the spin state, and others were set to the non-spin state. Photoexcited sites were randomly selected as adjacent Co1–W pairs with non-spin states, based on the specific ratio obtained from the experimental results. The number of magnetically coupled bonds was then calculated as the number of bonds between the spin-state centres, and the final value was obtained by averaging the results over 100 trials.

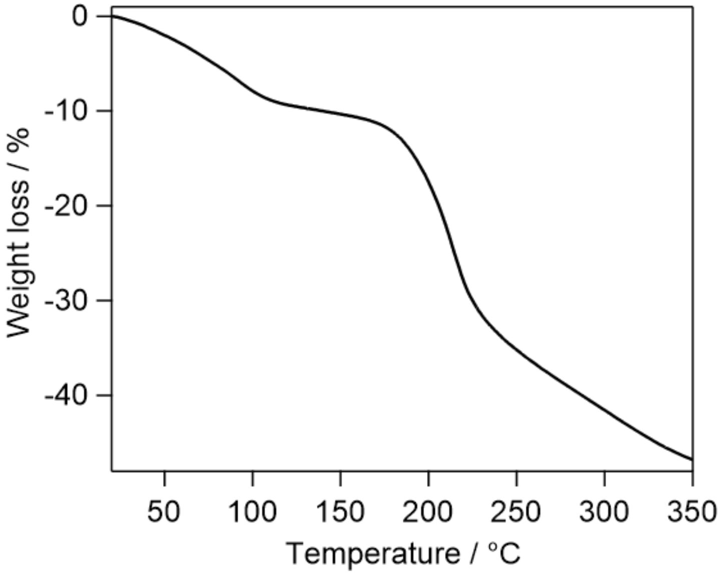

Figure S1 TG measurement of **1** under a scan rate of 5 K min^{-1}.

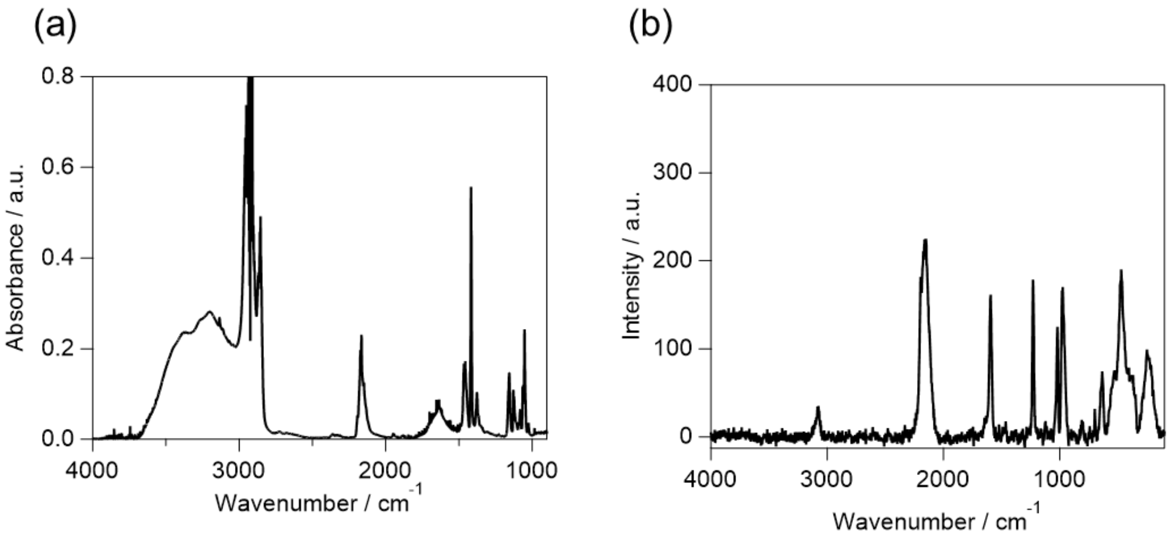

Figure S2 (a) IR and (b) Raman spectra of **1** at room temperature. The saturated peaks at approximately 3000 cm^{-1} in the IR spectrum were derived from liquid paraffin.

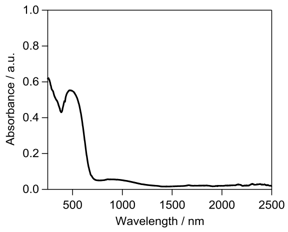

Figure S3 UV-vis-NIR spectrum of **1** at room temperature.

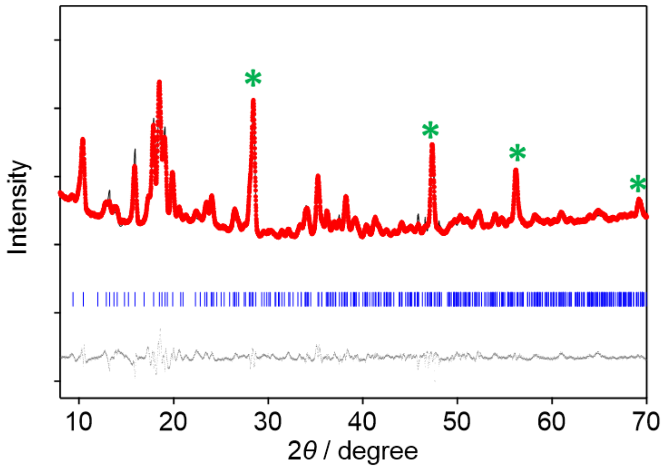

Figure S4 PXRD pattern and results of Rietveld analysis for **1** at room temperature. The red dots, black line, blue bar, and grey dots represent the experimental data, calculated pattern, Bragg diffractions of the target compound, and residual curve, respectively. The green asterisks indicate the standard Si peaks.

Table S1 Crystallographic data from Rietveld analysis for the PXRD pattern of **1**.

Crystal system	Tetragonal
Space group	*P4*/*mmm* (#123)
a / Å	18.9183(4)
c / Å	7.3793(3)
Z	2
V / Å^3	2641.05(14)
R_{wp}/ R_p	0.0248 / 0.0176
S	4.6880
T / K	293 (1)

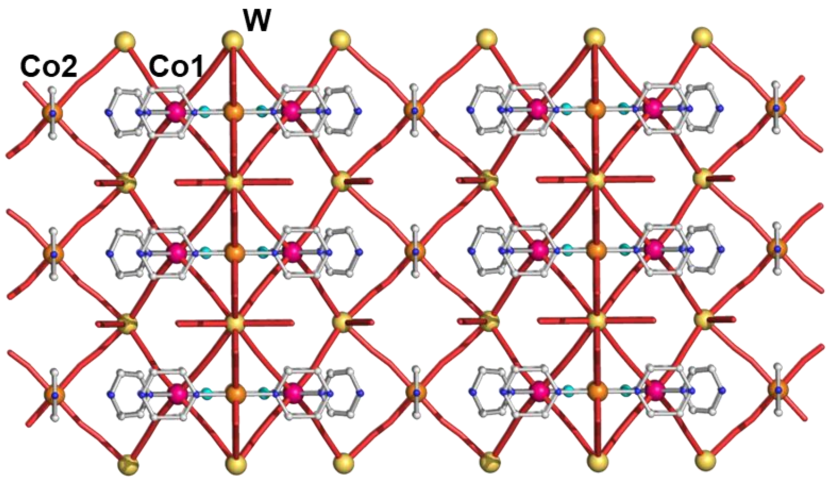

Figure S5 Crystal structure of **1** viewed from the *a*-axis. The pink, orange, yellow, and cyan spheres indicate Co1, Co2, W, and O respectively. The red line represents the networks cyanido-bridged by Co and W.

Table S2 Continuous shape measure (CSM) analysis for the $[W(CN)_8]^{n-}$ site of **1**.

CSM parameters			Geometry
SAPR-8	TDD-8	BTPR-8	
2.603	2.674	1.116	BTPR-8

*CSM parameters

CSM SAPR-8: parameter related to the square antiprism of D_{4d} symmetry

CSM BTPR-8: parameter related to the bicapped trigonal prism of C_{2v} symmetry

CSM TDD-8: parameter related to the dodecahedron of D_{2d} symmetry

CSM = 0 means the ideal geometry, and higher values mean distortion from the ideal geometry

----

S H A P E v2.1 Continuous Shape Measures calculation

(c) 2013 Electronic Structure Group, Universitat de Barcelona

Contact: llunell@ub.edu

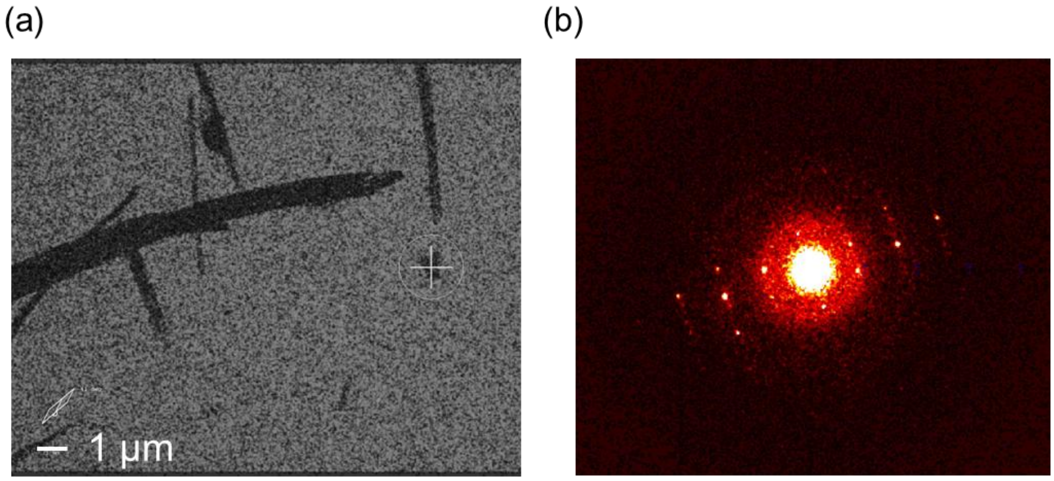

Figure S6 (a) Crystal and (b) diffraction picture of **1** obtained from MicroED measurements.

Table S3 Crystallographic parameters obtained from the MicroED experiment.

Refined formula	$C_{32.2}H_{16.2}Co_3N_{24.1}O_{1.9}W_2$
Crystal system	Tetragonal
Space group	*P*4/*mmm* (#123)
a / Å	18.409(5)
c / Å	7.2194(12)
Z	2
V / Å^3	2446.5 (10)
Crystal size / μm^3	0.9 × 0.8 × 0.1
Crystal color	Red needle
R_{int}	0.2089
R_1/ wR_2	0.1726 / 0.4537
T / K	293

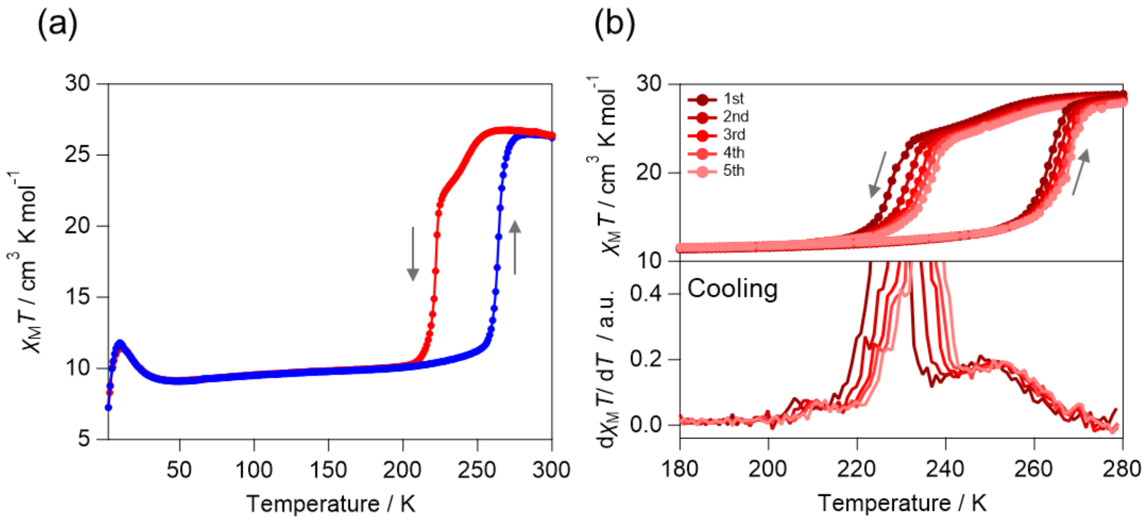

Figure S7 Temperature dependence of the χ_MT products of **1** under 5000 Oe during cooling (red circle) heating (blue circle) (a) in the range of 2–300 K and (b) in the range of 180–280 K with five cycles.

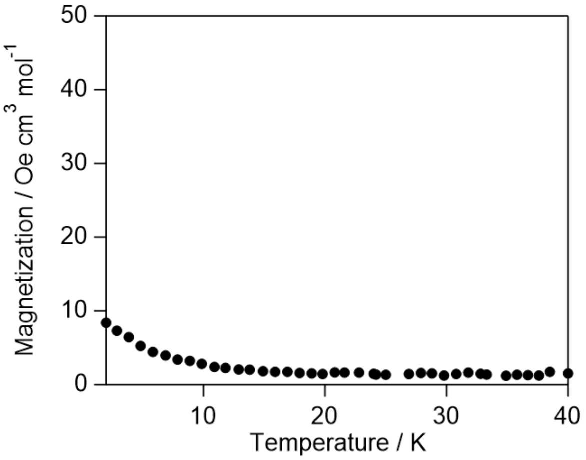

Figure S8 FCM curve of the LT phase of **1** under 20 Oe.

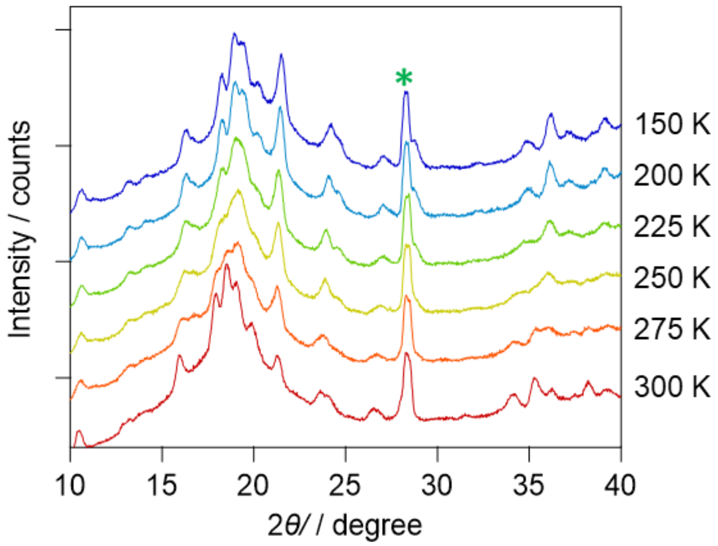

Figure S9 Variable-temperature PXRD patterns in the range of 10º–40º. The green asterisk indicates the Si peak.

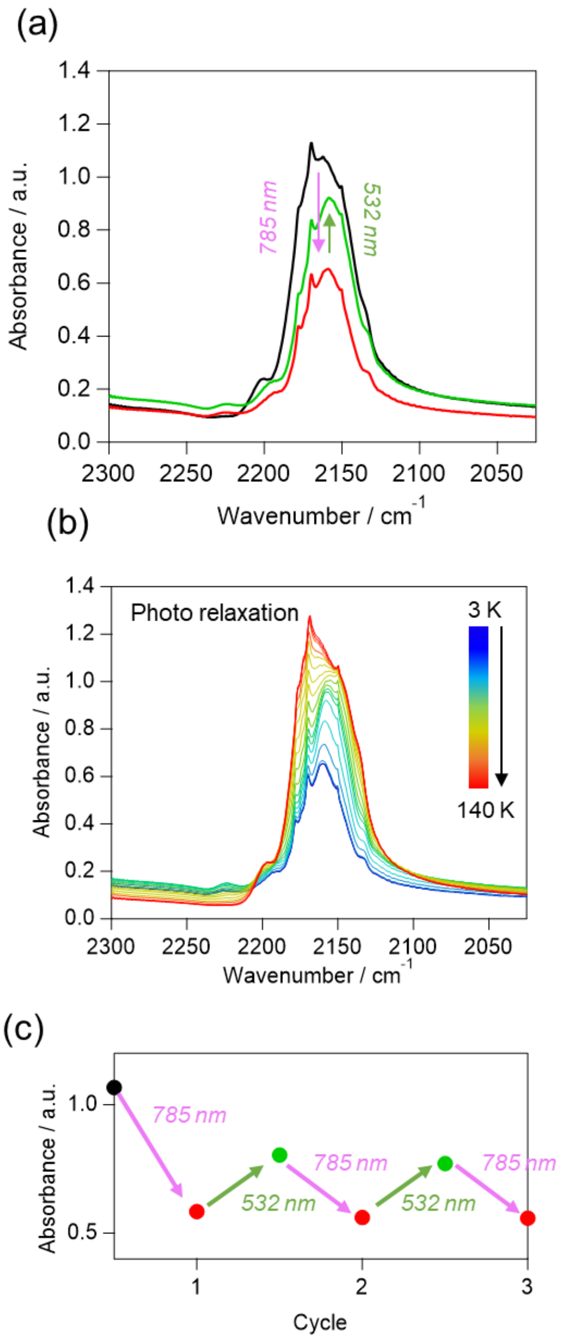

Figure S10 (a) IR spectra before and after photoirradiation. Black, red, and blue lines indicate the LT phase, the PI1 phase irradiated to the LT phase (785 nm, 220 mW cm^{-2}, 15 min.), and the PI2 phase irradiated to the PI1 phase (532 nm, 260 mW cm^{-2}, 15 min.), respectively. (b) Thermal-relaxed IR spectra of the PI1 phase of **1**. (c) Photoreversibility of IR spectra at 2160 cm^{-1} in three cycles.

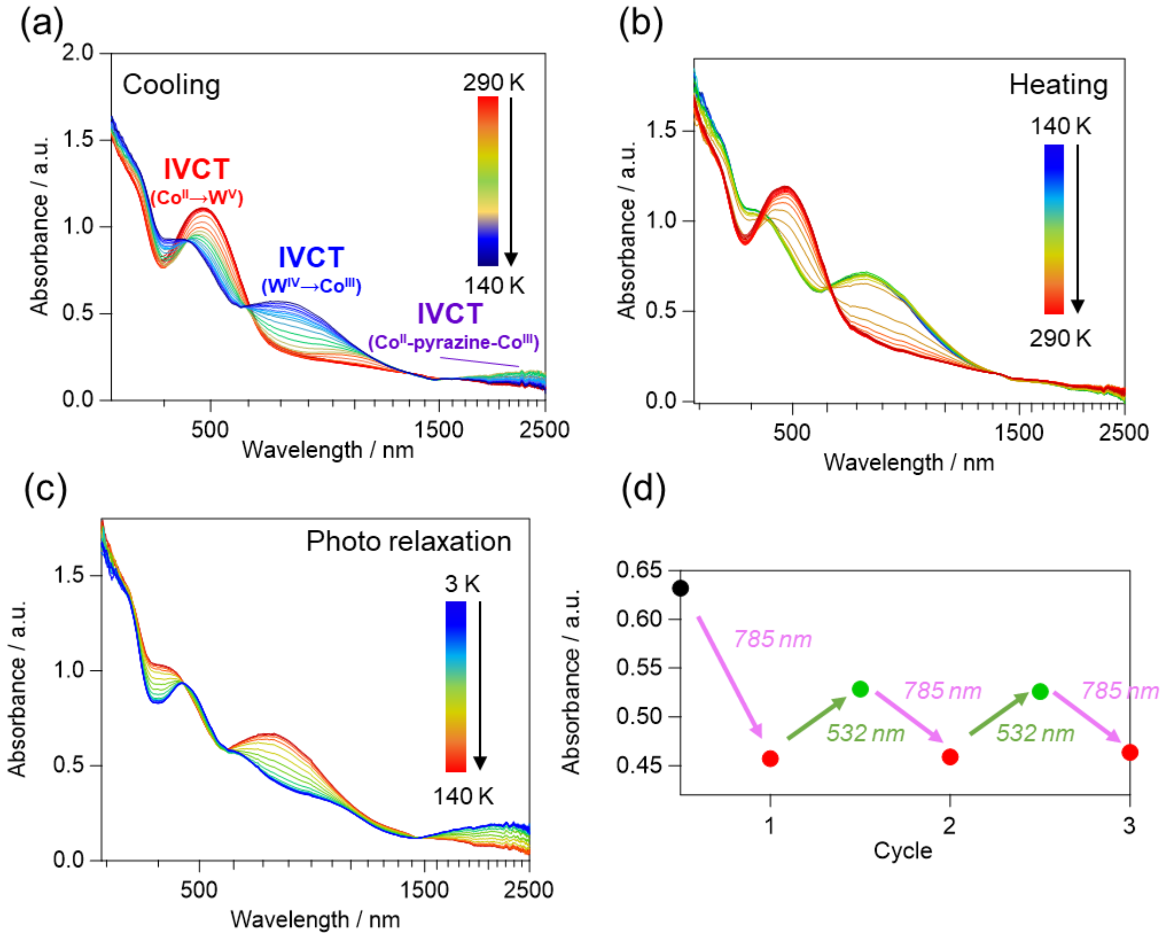

Figure S11 (a) Variable-temperature UV-vis-NIR spectra on (a) cooling and (b) heating process. (b) UV-vis-NIR spectra before and after photoirradiation. (c) Thermal-relaxed UV-vis-NIR spectra of the PI1 phase of **1** (785 nm, 220 mW cm^{-2}, 15 min.). (d) Photoreversibility of UV-vis-NIR spectra at 760 nm in three cycles.

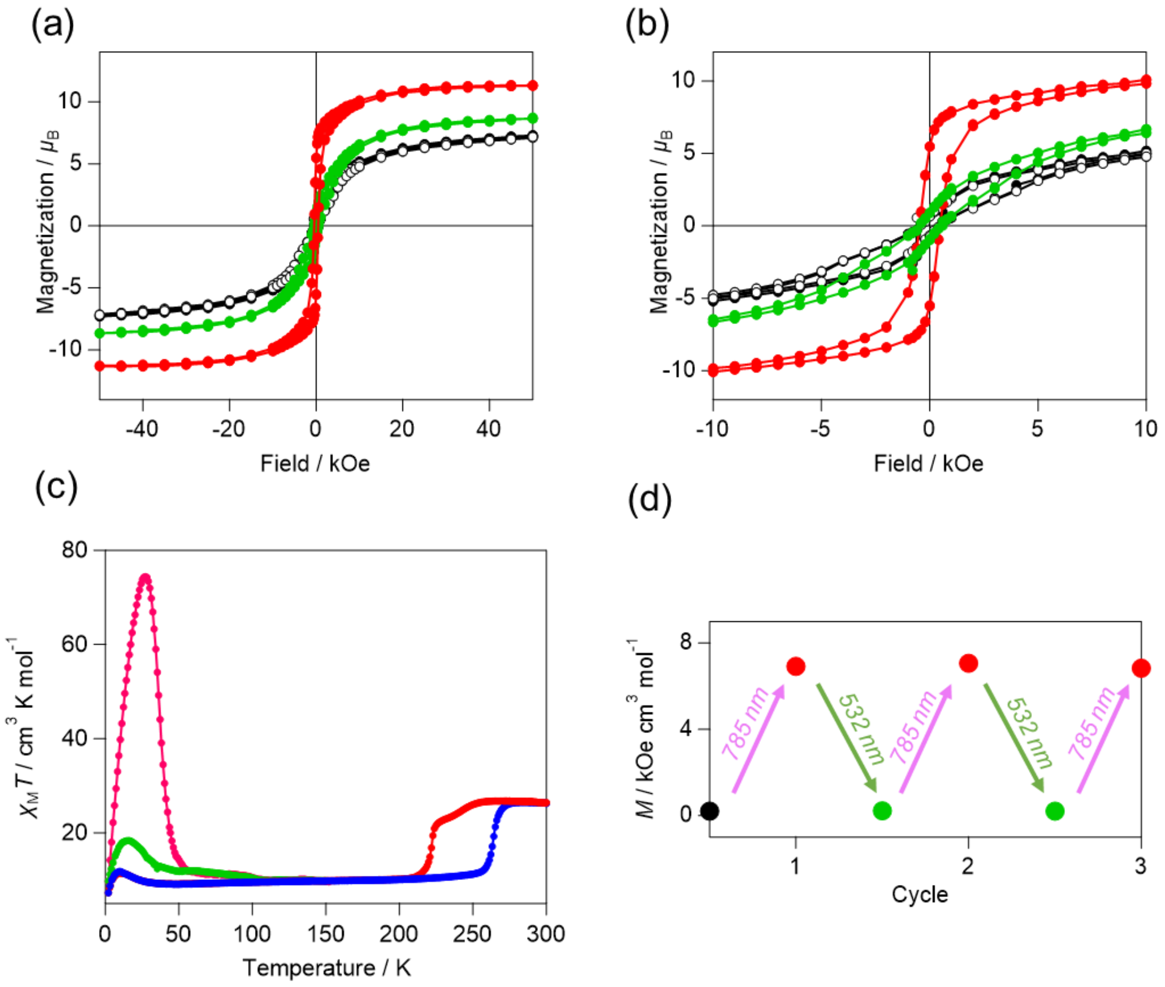

Figure S12 Photomagnetic measurements of **1**. (a), (b) M–H hysteresis curves at 3 K in the ranges from (a) −50 to 50 kOe and (b) −10 to 10 kOe. The black, red, unfilled black, and green circles indicate before and after photoirradiation (785 nm, 160 mW cm^{-2}, 10 min), after thermal annealing to 150 K, and after photoirradiation (532 nm, 110 mW cm^{-2}, 10 min) to the PI1 phase. (c) $\chi_M T$–T plots with the PI1 (pink) and PI2 (green) phases. (d) Photoreversibility confirmed by the magnetisation values at 3 K under 100 Oe.

[S1] J. G. Leipoldt, L. D. C. Bok, P. J. Cilliers, *Z. Anorg. Allg. Chem.* **1974**, *407*, 350–352.

[S2] L. D. C. Bok, J. G. Leipoldt, S. S. Basson, *Z. Anorg. Allg. Chem.* **1975**, *415*, 81–83.

[S3] S. Breda, I. D. Reva, L. Lapinski, M. J. Nowak, R. Fausto, *J. Mol. Struct.* **2006**, *786*, 193–206.

[S4] S. Ito, F. J. White, E. Okunishi, Y. Aoyama, A. Yamano, H. Sato, J. D. Ferrara, M. Jasnowskie, M. Meyer, *Cryst. Eng. Comm.* **2021**, *23*, 8622-8630.

[S5] G. M. Sheldrick, *Acta Cryst. A* **2015**, *71*, 3-8.

[S6] G. M. Sheldrick, *Acta Cryst. C* **2015**, *71*, 3-8.

[S7]O. V. Dolomanov, L. J. Bourhis, R. J. Gildea, J. A. K. Howard, H. Puschmann, *J. Appl. Cryst.* **2009**, *42*, 339-341.
